# Brusatol provokes a rapid and transient inhibition of Nrf2 signaling and sensitizes mammalian cells to chemical toxicity—implications for therapeutic targeting of Nrf2

**DOI:** 10.1016/j.freeradbiomed.2014.11.003

**Published:** 2015-01

**Authors:** Adedamola Olayanju, Ian M. Copple, Holly K. Bryan, George T. Edge, Rowena L. Sison, Min Wei Wong, Zheng-Quan Lai, Zhi-Xiu Lin, Karen Dunn, Christopher M. Sanderson, Ahmad F. Alghanem, Michael J. Cross, Ewa C. Ellis, Magnus Ingelman-Sundberg, Hassan Z. Malik, Neil R. Kitteringham, Christopher E. Goldring, B. Kevin Park

**Affiliations:** a MRC Centre for Drug Safety Science, Department of Molecular and Clinical Pharmacology, Institute of Translational Medicine, University of Liverpool, Liverpool, UK; b School of Chinese Medicine, Chinese University of Hong Kong, Hong Kong, People׳s Republic of China; c Department of Cellular and Molecular Physiology, Institute of Translational Medicine, University of Liverpool, Liverpool, UK; d Unit for Transplantation Surgery, Department of Clinical Science, Intervention, and Technology, Karolinska University Hospital Huddinge, Stockholm, Sweden; e Section of Pharmacogenetics, Department of Physiology and Pharmacology, Karolinska Institute, Stockholm, Sweden; f Department of Hepatobiliary Surgery, Aintree University Hospital NHS Foundation Trust, Liverpool, UK

**Keywords:** AMC, 7-amino-4-methylcoumarin, CDDO-Me, methyl-2-cyano-3,12-dioxooleana-1, 9(11)dien-28-oate, DNCB, 2,4-dinitrochlorobenzene, Gclm, glutamate–cysteine ligase regulatory subunit, HIF-1α, hypoxia-inducible factor 1α, IAA, iodoacetamide, Keap1, Kelch-like ECH-associated protein 1, NAPQI, *N*-acetyl-*p*-benzoquinone imine, NEM, *N*-ethylmaleimide, Nqo1, NAD(P)H dehydrogenase quinone 1, Nrf2, nuclear factor erythroid 2-related factor 2, RT-qPCR, real-time quantitative PCR., Brusatol, Nrf2, Keap1, Toxicity, Chemical stress, Cell defense, Hepatocyte, Free radicals

## Abstract

The transcription factor Nrf2 regulates the basal and inducible expression of a battery of cytoprotective genes. Whereas numerous Nrf2-inducing small molecules have been reported, very few chemical inhibitors of Nrf2 have been identified to date. The quassinoid brusatol has recently been shown to inhibit Nrf2 and ameliorate chemoresistance in vitro and in vivo. Here, we show that brusatol provokes a rapid and transient depletion of Nrf2 protein, through a posttranscriptional mechanism, in mouse Hepa-1c1c7 hepatoma cells. Importantly, brusatol also inhibits Nrf2 in freshly isolated primary human hepatocytes. In keeping with its ability to inhibit Nrf2 signaling, brusatol sensitizes Hepa-1c1c7 cells to chemical stress provoked by 2,4-dinitrochlorobenzene, iodoacetamide, and *N*-acetyl-*p*-benzoquinone imine, the hepatotoxic metabolite of acetaminophen. The inhibitory effect of brusatol toward Nrf2 is shown to be independent of its repressor Keap1, the proteasomal and autophagic protein degradation systems, and protein kinase signaling pathways that are known to modulate Nrf2 activity, implying the involvement of a novel means of Nrf2 regulation. These findings substantiate brusatol as a useful experimental tool for the inhibition of Nrf2 signaling and highlight the potential for therapeutic inhibition of Nrf2 to alter the risk of adverse events by reducing the capacity of nontarget cells to buffer against chemical and oxidative insults. These data will inform a rational assessment of the risk:benefit ratio of inhibiting Nrf2 in relevant therapeutic contexts, which is essential if compounds such as brusatol are to be developed into efficacious and safe drugs.

## Highlights

• Nrf2 regulates the expression of numerous cytoprotective genes.
• We show that the quassinoid brusatol rapidly and transiently inhibits Nrf2 signaling.
• Brusatol sensitizes mammalian cells to chemical stress provoked by electrophiles.
• Brusatol inhibits Nrf2 independent of its key regulatory mechanisms.
• Therapeutic inhibition of Nrf2 could enhance drug-induced adverse effects.

## Introduction

The redox-sensitive transcription factor nuclear factor erythroid 2-related factor 2 (Nrf2)^2^ plays a critical role in the regulation of cellular defense against chemical and oxidative stress [1]. The activity of Nrf2 is primarily governed by its physical and functional interaction with the cytosolic repressor Kelch-like ECH-associated protein 1 (Keap1), which facilitates the ubiquitination and subsequent proteasomal degradation of Nrf2 via the Cullin 3 ubiquitin ligase complex [2,3]. Upon exposure to chemical or oxidative stresses, the ability of Keap1 to repress Nrf2 is disrupted, leading to the latter׳s accumulation and translocation to the nucleus, where Nrf2 induces the transcription of a battery of cytoprotective genes encoding redox-balancing proteins, phase II detoxification enzymes, and drug transporters [4]. In doing so, Nrf2 promotes the maintenance of cellular homeostasis under stress conditions. Exemplifying the above, transgenic Nrf2-knockout mice demonstrate enhanced susceptibility to various drug-induced toxicities, including acetaminophen hepatotoxicity [5,6], cisplatin nephrotoxicity [7], and bleomycin lung fibrosis [8], whereas genetic knockdown of Keap1 confers a protective phenotype [9].

Recently, a number of studies have demonstrated a link between oncogenesis and mutations in the Keap1 and/or Nrf2 genes that result in the constitutive activation of Nrf2 [10]. In keeping with its ability to protect against chemical and oxidative stress, the constitutive activation of Nrf2 has also been shown to contribute to the development of drug resistance in cancer cell lines and tissues [11]. As a result, there is a burgeoning interest in the therapeutic potential of inhibiting Nrf2 as a strategy for overcoming chemoresistance. A barrier to this goal is the paucity of small-molecule inhibitors of Nrf2 that have been described to date, whereas the consequences for nontarget cell health of inhibiting Nrf2, particularly in the context of cytotoxic cancer therapy, have yet to be fully considered.

The quassinoid brusatol, isolated from the *Brucea javanica* plant, has recently been shown to inhibit Nrf2 signaling, reduce tumor burden, and ameliorate chemoresistance in both in vitro and in vivo cancer models [12–14]. Here, using Hepa-1c1c7 hepatoma cells and freshly isolated primary human hepatocytes, we demonstrate that brusatol provokes a rapid and transient depletion of Nrf2 protein, through a posttranscriptional mechanism that is independent of Keap1, the proteasomal and autophagic protein degradation systems, and protein kinase signaling pathways that are known to regulate Nrf2. In keeping with its ability to inhibit Nrf2 signaling, we show that brusatol sensitizes Hepa-1c1c7 cells to chemical stress provoked by 2,4-dinitrochlorobenzene (DNCB), iodoacetamide (IAA), and *N*-acetyl-*p*-benzoquinone imine (NAPQI), the hepatotoxic metabolite of acetaminophen. Therefore, whereas brusatol is a valuable experimental tool for inhibiting Nrf2, the benefit:risk ratio of its therapeutic use should be considered in light of the potential for enhanced sensitivity of nontarget cells to endogenous and exogenous chemical and oxidative insults.

## Materials and methods

### Materials

Brusatol was extracted and purified from fractions generated from dried plant material of *Fructus Bruceae* using Diaion HP-20, Diaion HP-20ss, and Sephadex LH-20 column chromatography and purified using a C18 semipreparative HPLC column (Alltima C18 column, 10×250 mm, 5 µm). The structure of brusatol was confirmed by NMR using a Bruker NMR spectrometer (400 MHz) with trimethylsilane as the internal standard. The structure was further confirmed by APCI–MS using an Agilent HP 1100 series SL Trap MSD. Methyl 2-cyano-3,12-dioxooleana-1,9(11)dien-28-oate (CDDO-Me) was kindly provided by Dr. Michael Wong and Professor Paul O’Neill (Department of Chemistry, University of Liverpool). All other materials were obtained from Sigma–Aldrich (UK).

### Hepa-1c1c7 cell culture

Mouse Hepa-1c1c7 hepatoma cells were maintained in Dulbecco׳s modified Eagle׳s medium supplemented with 584 mg/L l-glutamine, 10% (v/v) heat-inactivated fetal bovine serum (FBS; Biowest, France), 100 U/ml penicillin, and 100 µg/ml streptomycin, at 37 °C in a 5% CO_2_ humidified environment.

### Primary human hepatocyte isolation and culture

Liver tissue was obtained from the Liver Cell Lab at the Karolinska University Hospital (Huddinge, Sweden) or Aintree University Hospital (Liverpool, UK) by qualified medical staff, with donor informed consent following local ethical and institutional guidelines. The liver tissue used in this study was obtained from four patients (PHH1–4, see Supplementary Table S1 for details) undergoing planned liver resection for various indications. Immediately after removal from the patient, excess healthy liver parenchyma was separated from the specimen and placed in cold Eagle׳s minimum essential medium and transported to the laboratory on ice. Tissue dissociation and hepatocyte isolation were performed by using a two-step collagenase perfusion procedure, essentially as described previously [15]. The cells were counted and a Trypan blue exclusion test was used to calculate viability. The cell suspension was diluted to the required density in William׳s medium E without phenol red, supplemented with 25 mM Hepes and 2 mM l-glutamine, pH adjusted to 7.4 (modified William׳s medium E) supplemented with 10% FBS. Cells were seeded onto type I collagen-coated plates and cultured at 37 °C in a 5% CO_2_ humidified environment. After 3 h, the medium was replaced with fresh modified William׳s medium E not supplemented with FBS, and the cells were cultured for a further 16 h before commencement of experiments.

### Cell treatments

Cells were seeded into appropriate culture plates 24 h before the start of the experiments. All compounds were dissolved in dimethyl sulfoxide (DMSO) before addition to the cell culture medium, and the concentration of the vehicle was maintained at 0.5% (v/v) regardless of drug concentration.

### Immunoblotting

Cells were lysed in radioimmunoprecipitation assay buffer, and clarified whole-cell lysates were resolved by denaturing electrophoresis on 4–12% Novex Bis–Tris polyacrylamide gels (Life Technologies, UK). Separated proteins were transferred onto Hybond nitrocellulose membranes (GE Healthcare, UK), which were then blocked in Tris-buffered saline (TBS; pH 7.0) containing 0.1% Tween 20 and 10% nonfat milk (Bio-Rad). Blocked membranes were probed in TBS containing 0.1% Tween 20 and 2% nonfat milk supplemented with antibodies raised against Nrf2 (EP1808Y; Abcam, UK), β-actin (AC-15; Abcam), Keap1 (sc-15246; Santa Cruz Biotechnology, Germany), p62/SQSTM1 (P0067; Sigma–Aldrich), cyclin A (sc-751; Santa Cruz Biotechnology), hypoxia-inducible factor 1α (HIF-1α; 610959; BD Biosciences, UK), p53 (M7001; Dako, UK), survivin (sc-17779; Santa Cruz Biotechnology), phospho-p38 MAPK (4511S; Cell Signaling Technology), phospho-AKT (4060S; Cell Signaling Technology), phospho-ERK1/2 (4377S; Cell Signaling Technology), and phospho-SAPK (9251S; Cell Signaling Technology). Horseradish peroxidase-linked anti-rabbit (A9169; Sigma–Aldrich), anti-mouse (A9044; Abcam), and anti-goat (P0449; Dako) secondary antibodies were used as necessary. Immunoblots were visualized by enhanced chemiluminescence (PerkinElmer, UK) and exposed to Hyperfilm ECL (Amersham). Immunoreactive band volumes were quantified using TotalLab 100 software (Nonlinear Dynamics, UK) and normalized to β-actin.

### Measurement of cellular ATP content

Cell viability was measured using the CellTiter-Glo luminescence assay (Promega, UK), in accordance with the manufacturer׳s instructions.

### Real-time RT-PCR

Total RNA was extracted from cells and purified using an RNeasy Mini Kit from Qiagen, according to the manufacturer׳s instructions. cDNA was synthesized using the ImProm-II reverse transcription system (Promega) according to the manufacturer׳s instructions. Real-time quantitative PCR (RT-qPCR) analysis of the *Nrf2*, *Keap1*, NAD(P)H dehydrogenase quinone 1 (*Nqo1*), glutamate cysteine ligase regulatory subunit (*Gclm*), and glyceraldehyde-3-phosphate dehydrogenase (*Gapdh*) genes was performed on a ABI Prism 7000 real-time PCR instrument using Applied Biosystems (UK) SYBR green PCR master mix. *Gapdh* was used as a normalization control. Primer sequences are detailed in Supplementary Table S2.

### Real-time imaging of Nrf2–Venus

An SK-N-AS cell line stably expressing Nrf2–Venus under control of the endogenous human Nrf2 promoter region was kindly provided by Professor M. White (University of Manchester, UK). For live-cell imaging experiments, cells were seeded onto 35-mm glass-bottom dishes (Iwaki, Japan) before being exposed to 100 nM CDDO-Me for 1 h, followed by 300 nM brusatol. Nrf2–Venus expression was recorded at 2-min intervals by confocal microscopy, which was performed on a Zeiss LSM 780 microscope using a 20× plan-Apochromat objective. Cells were maintained in a humidified microscope stage incubator at 37 °C with 5% CO_2_ throughout the experiment.

### RNA interference

A small interfering RNA (siRNA) duplex targeted against mouse Keap1 and a scrambled, nontargeting control duplex (D-001210-03) were purchased from the Dharmacon siGENOME library. Cells were transfected for 48 h with 10 nM siRNA using Lipofectamine RNAiMAX (Life Technologies), according to the manufacturer׳s instructions.

### Measurement of proteasome activity

The human 20S proteasome was obtained from Enzo Life Sciences, and 1 µg of purified protein was preincubated for 30 min at 37 °C with the indicated concentrations of brusatol or MG132 in 50 mM Hepes (pH 7.8). Probe substrates for trypsin-like (Boc-LSTR-AMC, 50 µM; Sigma–Aldrich), chymotrypsin-like (Suc-LLVY-AMC, 50 µM; Enzo Life Sciences, UK), or caspase-like (Z-LLE-AMC, 400 µM; Enzo Life Sciences) activity were subsequently added and substrate cleavage was determined by quantification of 7-amino-4-methylcoumarin (AMC) fluorescence (excitation 360 nm, emission 460 nm) over 8 h and referenced to an AMC standard curve.

### Statistical analysis

Where applicable, data are expressed as the mean + standard deviation of the mean (SD) from *n*=3 independent experiments. The significance of differences between relevant data sets was assessed with an unpaired *t* test. A two-sided *P* value of ≤0.05 was considered statistically significant.

## Results

### Rapid and transient posttranscriptional inhibition of Nrf2 by brusatol

**Fig. 1:**
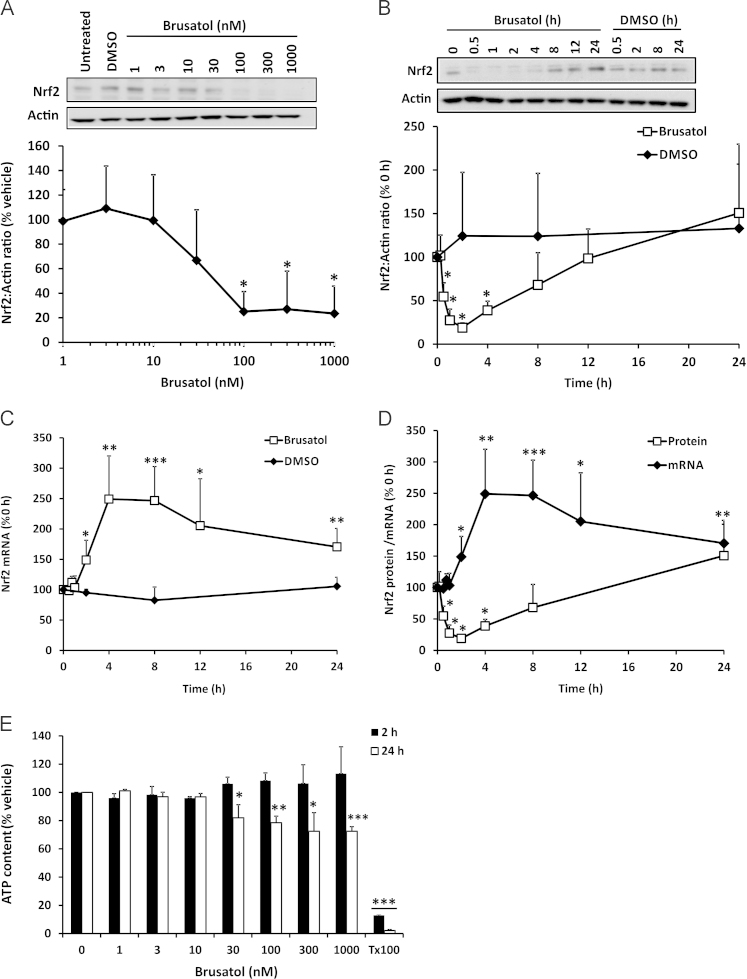
Rapid and transient posttranscriptional inhibition of Nrf2 by brusatol. (A and B) Nrf2 protein levels in whole-cell lysates prepared from Hepa-1c1c7 cells exposed to (A) the indicated concentrations of brusatol for 2 h or (B) 300 nM brusatol for the indicated times, as determined by immunoblotting. All blots shown are representative of three independent experiments. Nrf2 levels were quantified by densitometry, normalized to the loading control, β-actin, and expressed as a percentage of the Nrf2 level detected (A) in vehicle-exposed cells or (B) at 0 h. (C) *Nrf2* mRNA level in Hepa-1c1c7 cells exposed to 300 nM brusatol for the indicated times, as determined by RT-qPCR. *Nrf2* levels were normalized to the loading control, *Gapdh*, and expressed as a percentage of the *Nrf2* mRNA level detected at 0 h. (D) Superimposition of the time-course data for Nrf2 protein and mRNA presented in (B) and (C). (E) ATP content in Hepa-1c1c7 cells exposed to the indicated concentrations of brusatol for 2 or 24 h. ATP levels are expressed as a percentage of the ATP content of vehicle-exposed cells. All data represent the mean + SD of *n*=3 independent experiments. Statistical analyses were performed using an unpaired *t* test (*P<0.05, **P<0.01, ***P<0.001 vs vehicle or 0 h control).

To confirm that brusatol inhibits Nrf2, mouse Hepa-1c1c7 cells were exposed to a range of concentrations of the compound, and whole-cell Nrf2 levels were determined by immunoblotting. Within 2 h of exposure to cells, brusatol provoked the depletion of Nrf2, in a concentration-dependent manner (Fig. 1A). A detailed examination of the time course of the effect of brusatol on Nrf2 revealed a significant depletion of the transcription factor in as little as 30 min after exposure of the cells to brusatol (Fig. 1B). Notably, the inhibitory effect of brusatol on Nrf2 was transient in nature, as the resting level of Nrf2 was restored within 12 h of initial exposure to the compound (Fig. 1B). Moreover, there was evidence for a compensatory increase in Nrf2 protein, above the resting level, 24 h after exposure to brusatol (Fig. 1B). The rapid depletion of Nrf2 protein provoked by brusatol was not associated with a decrease in the level of Nrf2 mRNA (Fig. 1C), indicating that the effect on protein abundance is posttranscriptional. However, at later time points there was a significant increase in the level of Nrf2 mRNA, which reached a peak between 4 and 8 h after the initial exposure to brusatol (Fig. 1C). Superimposition of the mRNA and protein data indicated that the increase in Nrf2 mRNA immediately preceded the recovery of Nrf2 protein toward the resting level (Fig. 1D), implying that the transient nature of the Nrf2 inhibition provoked by brusatol is related to a compensatory induction of Nrf2 mRNA transcription that is stimulated by the compound itself and/or the rapid depletion of Nrf2 protein. Notably, concentrations of brusatol that caused the depletion of Nrf2 protein were minimally cytotoxic, as demonstrated by quantification of cellular ATP content (Fig. 1E). Taken together, these data demonstrate that brusatol potently induces the rapid and transient depletion of Nrf2 via a posttranscriptional mechanism.

### Functional consequences of Nrf2 inhibition by brusatol

**Fig. 2:**
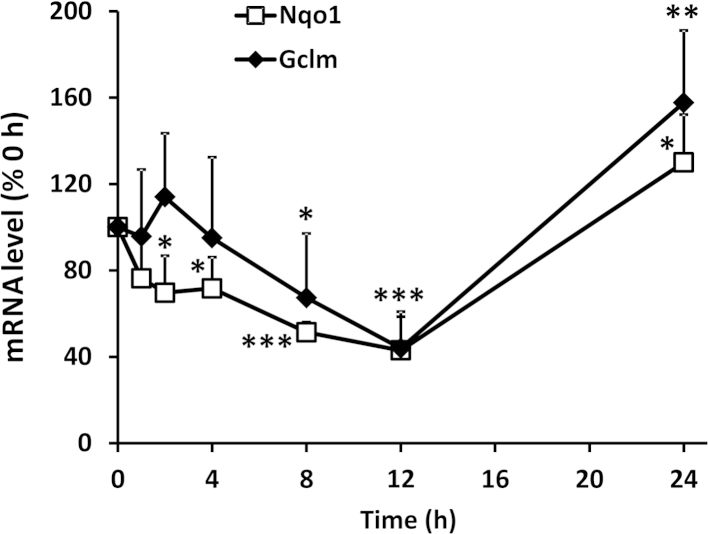
Functional consequences of Nrf2 inhibition by brusatol. The mRNA levels of the Nrf2 target genes *Nqo1* and *Gclm* in Hepa-1c1c7 cells exposed to 300 nM brusatol for the indicated times are shown, as determined by RT-qPCR. *Nqo1* and *Gclm* mRNA levels were normalized to the loading control, *Gapdh*, and expressed as a percentage of the *Nqo1* and *Gclm* levels detected at 0 h. All data represent the mean + SD of *n*=3 independent experiments. Statistical analyses were performed using an unpaired *t* test (*P<0.05, **P<0.01, ***P<0.001 vs 0-h control).

Consistent with the ability of brusatol to deplete Nrf2 protein, and the well-characterized role of Nrf2 in regulating the basal and inducible expression of cytoprotective genes, we detected significant time-dependent decreases in the mRNA levels of the classical Nrf2 target genes *Nqo1* and *Gclm* after exposure of Hepa-1c1c7 cells to 300 nM brusatol for up to 12 h, with evidence for a compensatory increase in the expression of these genes 24 h after exposure to the compound (Fig. 2). These results indicate that the rapid and transient depletion of Nrf2 provoked by brusatol causes a functional downregulation of Nrf2-sensitive cytoprotective processes.

### Inhibition of chemically induced Nrf2 by brusatol

**Fig. 3:**
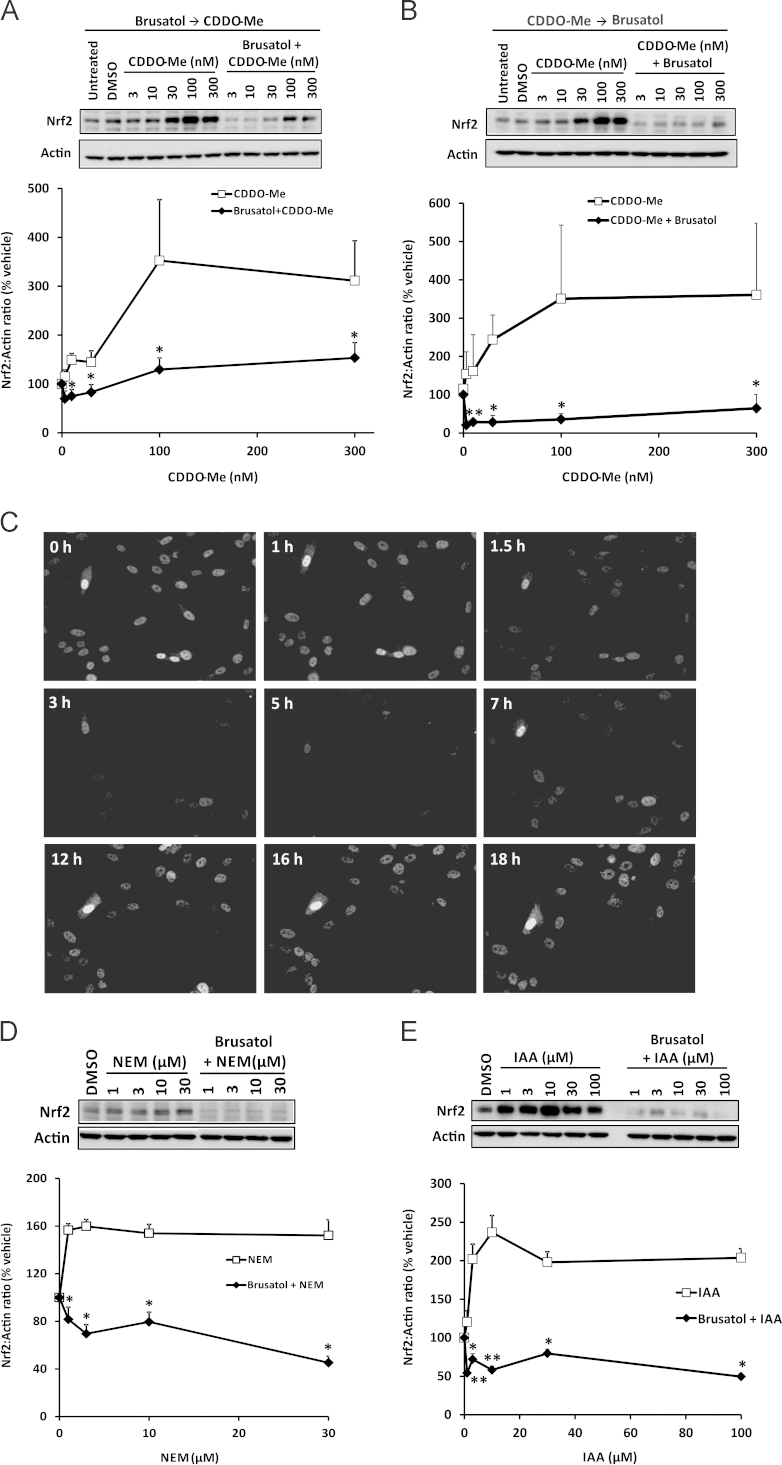
Inhibition of chemically induced Nrf2 by brusatol. (A and B) Nrf2 protein levels in whole-cell lysates prepared from Hepa-1c1c7 cells exposed to (A) vehicle or 300 nM brusatol for 2 h, followed by the indicated concentrations of CDDO-Me for 1 h, or (B) the indicated concentrations of CDDO-Me for 1 h, followed by 300 nM brusatol for 2 h, as determined by immunoblotting. (C) Nrf2–Venus levels in SK-N-AS cells exposed to 100 nM CDDO-Me for 1 h, followed by 300 nM brusatol for the indicated times. Still images were captured from the real-time data presented in Supplementary Video 1. (D and E) Nrf2 protein levels in whole-cell lysates prepared from Hepa-1c1c7 cells exposed to vehicle or 300 nM brusatol for 2 h, followed by the indicated concentrations of (D) NEM or (E) IAA for 1 h. All blots shown are representative of three independent experiments. Nrf2 levels were quantified by densitometry, normalized to the loading control, β-actin, and expressed as a percentage of the Nrf2 level detected in vehicle-exposed cells. Data represent the mean + SD of *n*=3 independent experiments. Statistical analysis was performed using an unpaired *t* test (*P<0.05, **P<0.01 vs CDDO-Me, IAA, or NEM alone).

**Fig. 4:**
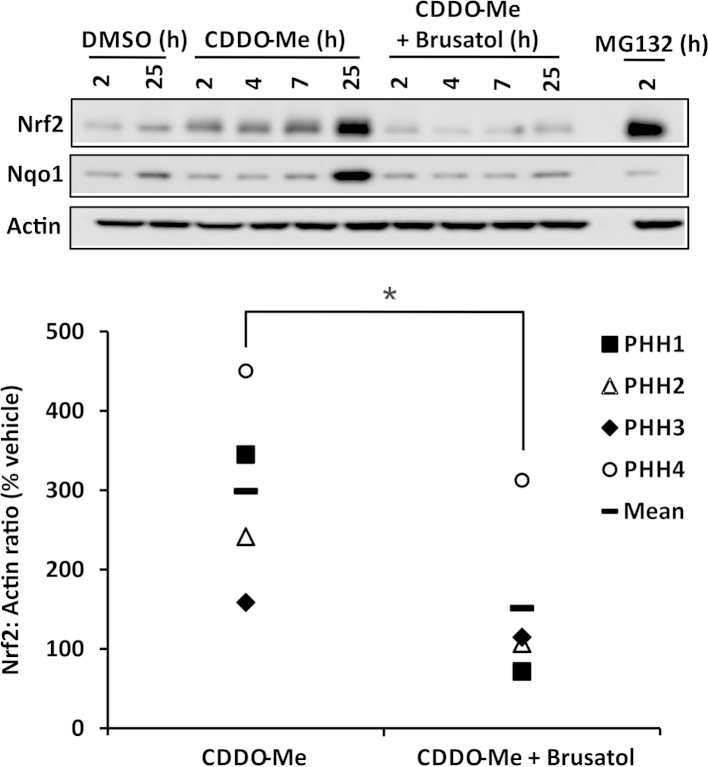
Inhibition of Nrf2 by brusatol in primary human hepatocytes. Nrf2 and Nqo1 protein levels in whole-cell lysates prepared from freshly isolated primary human hepatocytes exposed to 100 nM CDDO-Me for 1 h, followed by 100 nM brusatol for the remainder of the indicated times, are shown as determined by immunoblotting. The proteasome inhibitor MG132 was used as a positive control for Nrf2 accumulation. The blots shown are from samples derived from donor PHH1 and are representative of experiments performed using cells isolated from four donors (PHH1–4). Nrf2 levels in cells exposed to CDDO-Me and brusatol for a total of 25 h were quantified by densitometry, normalized to the loading control, β-actin, and expressed as a percentage of the Nrf2 level detected in vehicle-exposed cells. Statistical analysis was performed using an unpaired *t* test (*P<0.05 vs CDDO-Me alone).

A potential therapeutic application of an Nrf2 inhibitor such as brusatol is the downregulation of Nrf2 pathway components in cells harboring constitutively high levels of the transcription factor. Such a phenomenon is increasingly linked with the development of chemoresistance in cancer cells [11,16]. To examine the ability of brusatol to deplete elevated levels of Nrf2, we used the chemical inducers CDDO-Me (also known as bardoxlone methyl), *N*-ethylmaleimide (NEM), and IAA. Pretreatment of Hepa-1c1c7 cells with 300 nM brusatol for 2 h, followed by exposure to a range of concentrations of CDDO-Me for 1 h, markedly inhibited the ability of the latter compound to provoke Nrf2 accumulation (Fig. 3A). Moreover, brusatol was able to deplete Nrf2 when applied to cells in which the transcription factor had already accumulated after pretreatment with CDDO-Me (Fig. 3B). This finding was confirmed by real-time fluorescence imaging of human SK-N-AS neuroblastoma cells engineered to stably express Venus-tagged Nrf2 under control of an endogenous human promoter. In these cells, CDDO-Me provoked a substantial accumulation of Nrf2–Venus within 1 h, yet subsequent addition of brusatol stimulated a rapid and transient depletion of Nrf2–Venus (Fig. 3C and Supplementary Vedeo 1). To ensure that the above observations were not specific to CDDO-Me, we pretreated Hepa-1c1c7 cells with 300 nM brusatol for 2 h and stimulated Nrf2 accumulation by subsequent exposure to a range of concentrations of the electrophiles NEM or IAA for 1 h. Under these conditions, brusatol markedly attenuated the ability of NEM or IAA to induce Nrf2 (Figs. 3D and E). Consistent with these findings, and in a demonstration of the translational relevance of the data generated with Hepa-1c1c7 cells, brusatol was also shown to inhibit the accumulation of Nrf2 and its downstream target Nqo1 when applied to freshly isolated primary human hepatocytes in which the transcription factor had been induced via pretreatment with CDDO-Me (Fig. 4). Taken together, these data confirm that brusatol is capable of stimulating the depletion of elevated levels of Nrf2 in mammalian cells.

Supplementary material related to this article can be found online at doi:10.1016/j.freeradbiomed.2014.11.003.

The following is the Supplementary material related to this article Video 1.

Video 1 **Real-time imaging of Nrf2-Venus in SK-N-AS cells exposed to CDDO-Me and brusatol.** An SK-N-AS cell line, stably expressing Nrf2-Venus under control of the endogenous human Nrf2 promoter, was exposed to 100 nM CDDO-Me at 0 h, followed by 300 nM brusatol at 1 h. Nrf2-Venus expression was recorded at 2 min intervals by confocal microscopy. Video uploaded separately..

### Brusatol sensitizes mammalian cells to chemical stress

**Fig. 5:**
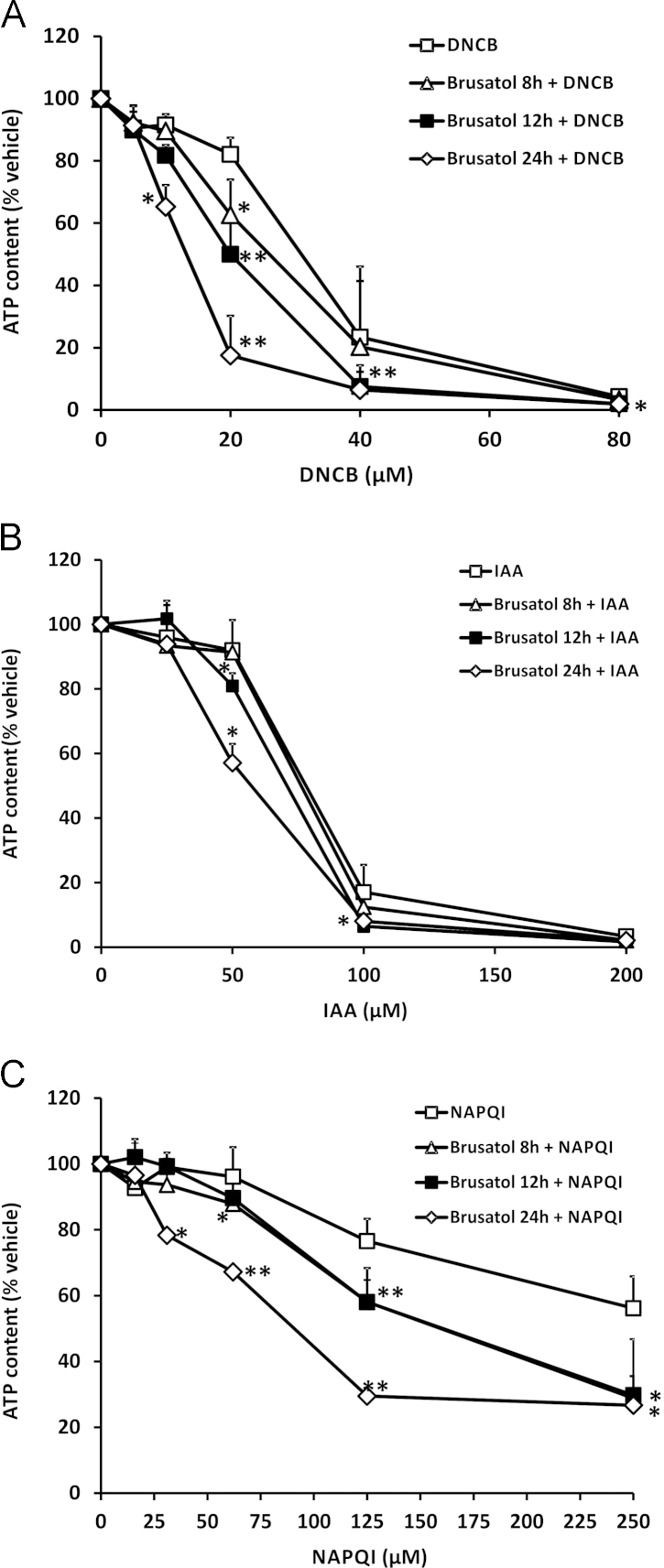
Brusatol sensitizes mammalian cells to chemical stress. ATP content in Hepa-1c1c7 cells exposed to 300 nM brusatol for the indicated times, followed by the indicated concentrations of (A) DNCB for 6 h, (B) IAA for 6 h, or (C) NAPQI for 12 h is shown. ATP levels are expressed as a percentage of the ATP content of vehicle-exposed cells. Data represent the mean + SD of *n*=3 independent experiments. Statistical analysis was performed using an unpaired *t* test (*P<0.05, **P<0.01 vs vehicle control).

In light of our data demonstrating the ability of brusatol to inhibit Nrf2 signaling, and given the well-defined role of Nrf2 in regulating the activity of cytoprotective processes in mammalian cells, we sought to test the hypothesis that brusatol-mediated depletion of Nrf2 would sensitize Hepa-1c1c7 cells to the deleterious effects of chemical stress provoked by model electrophiles. Indeed, pretreatment of cells with brusatol enhanced the cytotoxicity elicited by DNCB, IAA, and NAPQI (Figs. 5A–C). Notably, increasing the duration of the brusatol pretreatment period resulted in a greater potentiation of cytotoxicity in each case (Figs. 5A–C). Therefore, these data indicate that brusatol is capable of sensitizing mammalian cells to chemical stress.

### The inhibitory effect of brusatol is specific to Nrf2 but not dependent on the canonical mechanisms of Nrf2 degradation

**Fig. 6:**
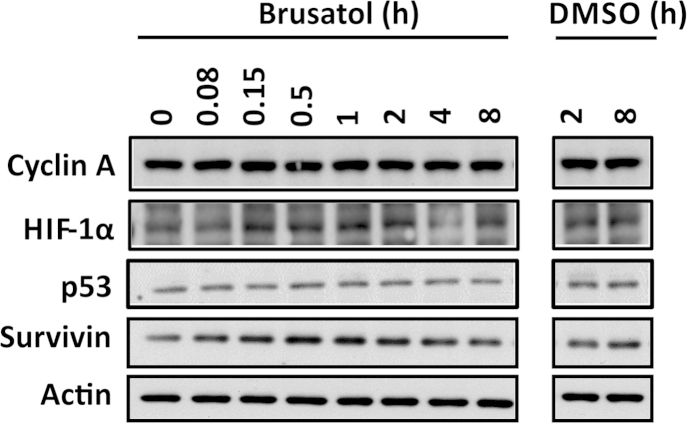
Brusatol does not provoke the general depletion of short-half-life proteins in Hepa-1c1c7 cells. Cyclin A, HIF-1α, p53, and survivin protein levels in whole-cell lysates prepared from Hepa-1c1c7 cells exposed to 300 nM brusatol for the indicated times are shown, as determined by immunoblotting. All blots shown are representative of three independent experiments.

An understanding of the mechanism(s) by which brusatol inhibits Nrf2 is critical to evaluating the benefit:risk ratio of the compound, and other Nrf2 inhibitors, in therapeutic contexts. We noted that brusatol and structurally related compounds have been shown previously to inhibit general protein synthesis at micromolar concentrations [17,18]. We also noted that, among the panel of proteins measured by Ren et al. [12] in their recent report, only c-Myc, which has a relatively short half-life of 20–30 min, was depleted in response to brusatol, albeit to a lesser degree than Nrf2. Given that Nrf2 also has a relatively short half-life (15–30 min in the absence of chemical or oxidative stimuli), we examined the effects of brusatol on other proteins with short half-lives, to determine the specificity of brusatol toward Nrf2. Importantly, brusatol had no discernible effect on the basal levels of cyclin A, HIF-1α, p53, or survivin under conditions that were associated with substantial Nrf2 depletion (Fig. 6). These data indicate that the inhibitory effect of brusatol is specific, and not a consequence of a broader effect on protein synthesis, at the nanomolar concentrations required to deplete Nrf2.

**Fig. 7:**
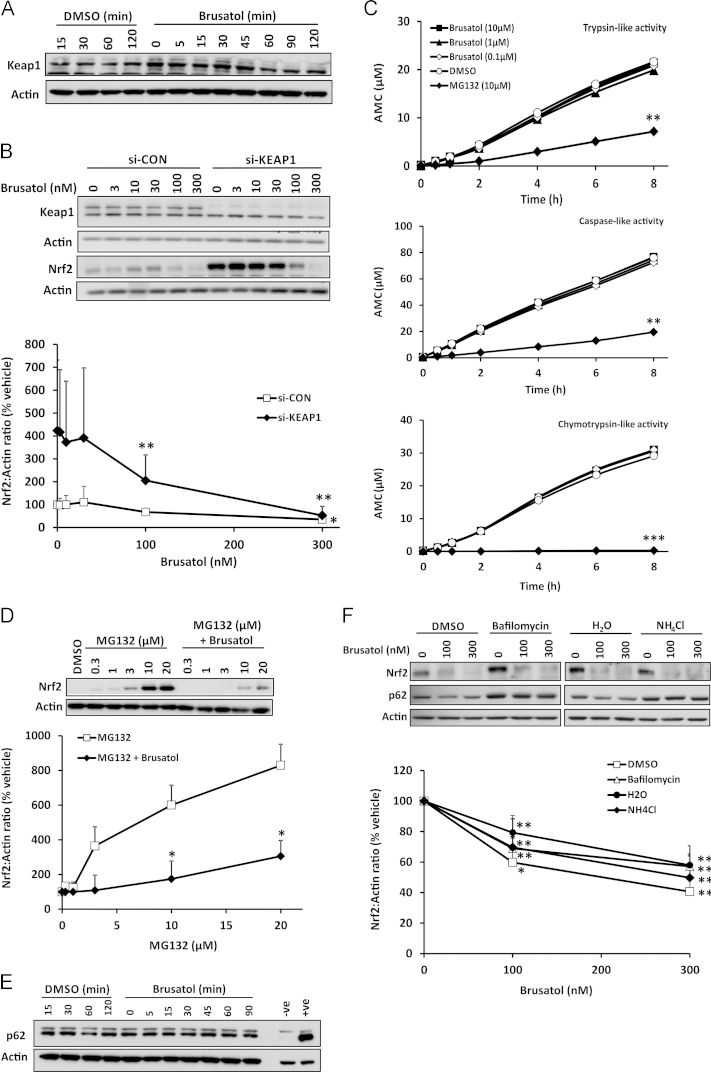
Inhibition of Nrf2 by brusatol is not dependent on the canonical mechanisms of Nrf2 degradation. (A) Keap1 protein levels in whole-cell lysates prepared from Hepa-1c1c7 cells exposed to 300 nM brusatol for the indicated times, as determined by immunoblotting. (B) Keap1 and Nrf2 protein levels in whole-cell lysates prepared from Hepa-1c1c7 cells transfected with a scrambled, nontargeting control siRNA (si-CON) or a Keap1-targeting siRNA (si-KEAP1) for 48 h, followed by exposure to the indicated concentrations of brusatol for 2 h. (C) Effects of incubation with the indicated concentrations of brusatol or the proteasome inhibitor MG132, for the indicated times, on the chymotrypsin (Suc-LLVY-AMC)-, trypsin (Boc-LSTR-AMC)-, and caspase (Z-LLE-AMC)-like activities of human 20S proteasome. (D) Nrf2 protein levels in whole-cell lysates prepared from Hepa-1c1c7 cells exposed to the indicated concentrations of MG132 for 1 h, followed by 300 nM brusatol for 2 h. (E) p62 protein levels in whole-cell lysates prepared from Hepa-1c1c7 cells exposed to 300 nM brusatol for the indicated times. Lysates prepared from mock-transfected (-ve) and p62-transfected (+ve) HEK293T cells were loaded as controls. (F) Nrf2 and p62 protein levels in whole-cell lysates prepared from Hepa-1c1c7 cells exposed to 30 nM bafilomycin A1 or 10 mM ammonium chloride (NH_4_Cl) for 16 h, followed by the indicated concentrations of brusatol for 2 h. All blots shown are representative of three independent experiments. Nrf2 levels were quantified by densitometry, normalized to the loading control, β-actin, and expressed as a percentage of the Nrf2 level detected in vehicle-exposed cells. Data represent the mean + SD of *n*=3 independent experiments. Statistical analysis was performed using an unpaired *t* test (*P<0.05, **P<0.01, ***P<0.001 vs vehicle control or MG132 alone).

Given that our data demonstrated an ability of brusatol to deplete Nrf2 protein in a rapid and posttranscriptional manner (Fig. 1D), we examined the dependence of brusatol׳s inhibitory action on the classical mechanisms of Nrf2 protein regulation, namely Keap1-mediated repression [3], and degradation via the proteasome [19] and autophagy [20] systems. After exposure of Hepa-1c1c7 cells to 300 nM brusatol for up to 2 h, conditions that were sufficient to cause maximal depletion of Nrf2 (Fig. 1C), there was no overt change in the level of Keap1 protein (Fig. 7A), indicating that the marked decrease in Nrf2 was not caused by a concomitant increase in the level of its cytosolic inhibitor. Furthermore, in cells transfected with Keap1-targeting siRNA, which depleted Keap1 protein by 90% and caused a substantial accumulation of Nrf2 (Fig. 7B), brusatol was able to deplete Nrf2 as efficiently as in cells transfected with a scrambled, nontargeting control siRNA (Fig. 7B). These data indicate that brusatol provokes the depletion of Nrf2 independent of Keap1.

To examine the potential for brusatol to directly augment the proteasomal degradation of Nrf2, we determined the effects of the compound on the chymotrypsin-, trypsin-, and caspase-like activities of human 20S proteasome in vitro. In each case, brusatol had no discernible effect on proteasome activity (Fig. 7C). Consistent with these findings, brusatol was able to deplete Nrf2 when added to cells in which Nrf2 levels had been increased via pretreatment with the proteasomal inhibitor MG132 (Fig. 7D). Therefore, brusatol appears to deplete Nrf2 via a process that does not involve its enhanced proteasomal degradation. A major alternative cellular mechanism of protein degradation is macroautophagy. Processing of p62 is commonly used as an indicator of augmented autophagy [21], and p62 has been shown to physically interact with Keap1 and direct the Keap1–Nrf2 complex to the autophagy machinery [20]. However, we found no evidence for a change in the level of p62 under conditions of maximal Nrf2 depletion by brusatol (Fig. 7E). Furthermore, although chemical inhibition of autophagy (by bafilomycin or ammonium chloride) stimulated an accumulation of p62, and to a lesser degree Nrf2, brusatol was still able to provoke the maximal depletion of Nrf2 under these conditions (Fig. 7F). Therefore, these data indicate that brusatol provokes the depletion of Nrf2 via a mechanism that is not dependent on Keap1 and the proteasomal and autophagic protein degradation systems.

### Inhibition of Nrf2 by brusatol is not dependent on the activation of p38 MAPK, AKT, ERK1/2, or JNK1/2 signaling

**Fig. 8:**
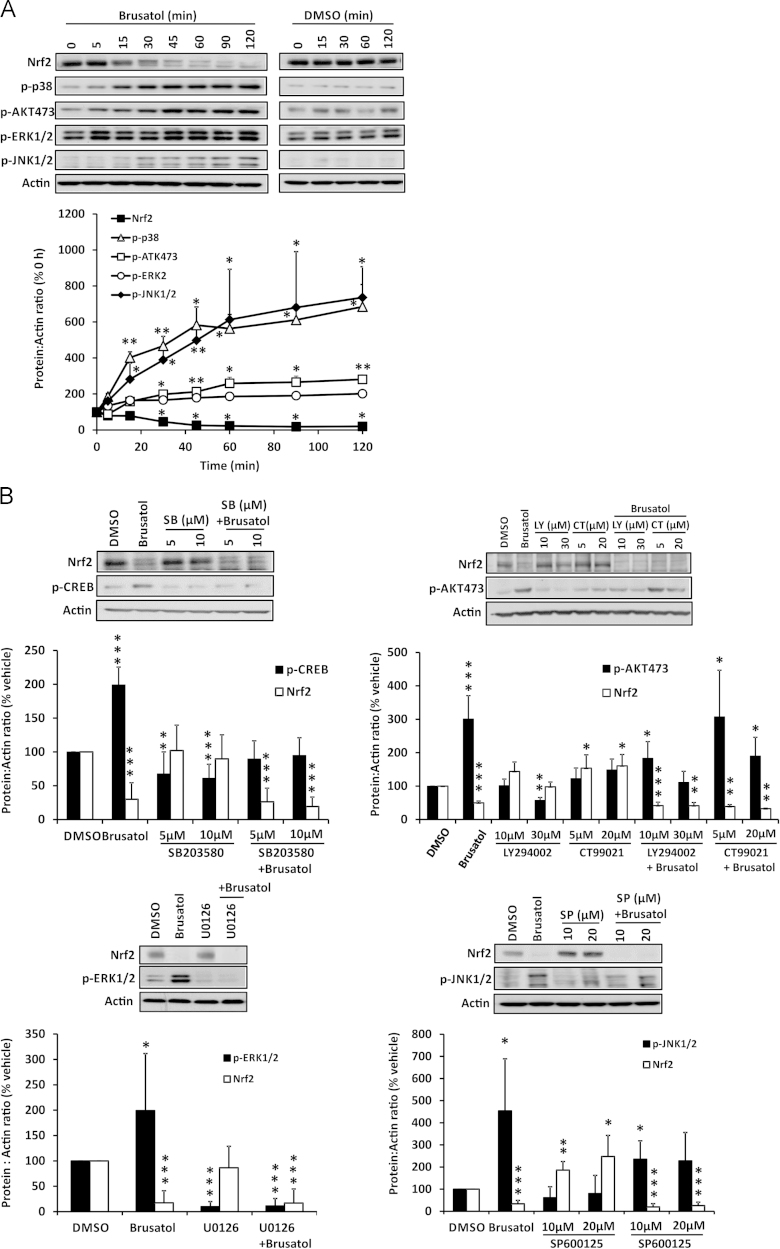
Inhibition of Nrf2 by brusatol is not dependent on the activation of p38 MAPK, AKT, ERK1/2, or JNK1/2 signaling. (A) Nrf2 and phospho-p38 MAPK, AKT, ERK1/2, and JNK1/2 levels in whole-cell lysates prepared from Hepa-1c1c7 cells exposed to 300 nM brusatol for the indicated times, as determined by immunoblotting. (B) Nrf2 protein levels in whole-cell lysates prepared from Hepa-1c1c7 cells exposed to the indicated concentrations of SB203580 (p38 MAPK), LY294002 (AKT), CT99021 (GSK3β), or SP600125 (JNK1/2) or 5 µM U0126 (ERK1/2) for 1 h, followed by 300 nM brusatol for 2 h. All blots shown are representative of three independent experiments. Nrf2 or protein kinase levels were quantified by densitometry, normalized to the loading control, β-actin, and expressed as a percentage of the Nrf2 or protein kinase level detected at 0 h or in vehicle-exposed cells. Data represent the mean + SD of *n*=3 independent experiments. Statistical analysis was performed using an unpaired *t* test (*P<0.05, **P<0.01, ***P<0.001 vs vehicle or 0 h control).

In addition to Nrf2, protein kinases are among the most sensitive cellular signaling components that are modulated by chemical perturbation. Therefore, we examined the ability of brusatol to alter the activity of several protein kinases that have been implicated in Nrf2 signaling. Notably, brusatol provoked a rapid increase in the phosphorylation of p38 MAPK, AKT, ERK1/2, and JNK1/2 in parallel with depletion of Nrf2 (Fig. 8A). However, the inhibitory effect of brusatol on Nrf2 was shown to be independent of these events, as preincubation of cells with the kinase inhibitors SB203580 (p38 MAPK), LY294002 (AKT), CT99021 (GSK3β), U0126 (ERK1/2), and SP600125 (JNK1/2) had no discernible effect on the ability of brusatol to deplete Nrf2 (Fig. 8B). Therefore, brusatol inhibits Nrf2 via a mechanism that is not dependent on the activation of p38 MAPK, AKT, ERK1/2, or JNK1/2 signaling. Taken together with our data demonstrating that brusatol׳s inhibitory effect on Nrf2 is independent of Keap1 and the proteasomal and autophagic protein degradation systems, these findings imply the involvement of a novel means of Nrf2 regulation in the mechanism of action of this compound.

## Discussion

To date, the majority of translational research on Nrf2 has focused on its role in protection against disease-inducing insults and the potential value of pharmacologically activating Nrf2 signaling in the prevention and/or treatment of various pathologies [22]. However, prompted by evidence that hyperactivation of Nrf2 signaling is associated with the progression of cancer and development of chemoresistance [11], researchers have recently begun to search for chemical inhibitors of Nrf2 and to examine their pharmacological mechanisms of action. Consistent with recent reports [12–14], here we have shown that the natural quassinoid brusatol provokes a rapid and transient depletion of Nrf2 protein, through a posttranscriptional mechanism, in mouse Hepa-1c1c7 hepatoma cells and freshly isolated primary human hepatocytes and sensitizes mammalian cells to the cytotoxic effects of model electrophiles. These findings verify brusatol as a valuable experimental tool for studying the cytoprotective functions of Nrf2, particularly when the genetic manipulation of Nrf2 is not suitable or favorable.

The development of chemoresistance is a barrier to the effective management of various forms of cancer [23]. In light of the recently established role of Nrf2 in drug resistance in cancer cell lines and tissues [11], there is much interest in the potential value of inhibiting Nrf2 to enhance the efficacy of established and novel chemotherapeutic agents. Zhang and colleagues recently demonstrated the ability of brusatol to ameliorate chemoresistance in both in vitro and in vivo cancer models [12–14]. However, given the established role of Nrf2 as a key regulator of cell defense against endogenous and exogenous chemical and oxidative insults, it is possible that indiscriminate inhibition of Nrf2 may sensitize nontarget cells to the cytotoxic effects of cancer drugs, thereby exacerbating adverse effects. Such a concept is supported by our finding that brusatol enhances the cytotoxic effects of model electrophiles toward mammalian cells in vitro. Analogous to the drive to target chemotherapy based on the cell type and/or molecular landscape of specific cancers, an ability to direct the pharmacological inhibition of Nrf2 by brusatol and other compounds to relevant cells may provide an opportunity to enhance therapeutic efficacy and circumvent the potential for enhancement of off-target toxicity. Even if such an approach is realized, the risk:benefit ratio of inhibiting Nrf2 needs greater consideration before it can be developed into a viable strategy for the treatment of chemoresistant forms of cancer and other diseases in which aberrant Nrf2 signaling plays a role.

Although brusatol and structurally related compounds can serve as general protein synthesis inhibitors at micromolar concentrations [17,18], the lack of effect of brusatol on the levels of other proteins [12], including those with short (cyclin A, HIF-1α, p53, and survivin) and long (Keap1, p62, and actin) half-lives studied here, indicates that the depletion of Nrf2 provoked by nanomolar concentrations of brusatol is specific and not a consequence of a broader effect on protein synthesis. Previous work has demonstrated that brusatol is able to deplete Nrf2 in cancer cell lines harboring wild-type or mutated Keap1 [12]. This finding indirectly implied that the inhibitory action of brusatol is not dependent on Keap1. Here, we have provided direct evidence for this notion, through siRNA knockdown of Keap1, which had no discernible effect on the ability of brusatol to deplete Nrf2 in a rapid and transient manner. These data highlight the potential value of brusatol as an inhibitor of Nrf2 signaling in contexts, such as cancer [11], in which the negative regulation of Nrf2 by Keap1 has been circumvented. We have also demonstrated that the classical cellular mechanisms of protein degradation, namely the ubiquitin–proteasome system and autophagy, are dispensable for brusatol׳s inhibitory action toward Nrf2. Although these observations do provide valuable insights into the means by which brusatol downregulates Nrf2 signaling, and imply the involvement of a novel means of Nrf2 regulation in the pharmacodynamic effects of this compound, further investigation will be required to reveal the detailed underlying mechanism(s). Recently, it has been suggested that an as-yet unidentified molecular process represses the translation of Nrf2 mRNA within the open reading frame of the gene [24]. It is possible that brusatol could temporarily augment this translational repression. Indeed, this would account for our observation that the rapid and substantial depletion of Nrf2 protein provoked by brusatol is not preceded by a discernible decrease in the level of Nrf2 mRNA. Further work is needed to test this hypothesis.

A notable feature of brusatol׳s inhibitory action toward Nrf2 is the transient nature of its effect and the compensatory increase in the level of Nrf2 and its target genes 24 h after exposure to the compound. A similar finding has been documented in A549 cells by Ren et al. [12]. Knowledge of the chemical and/or molecular mechanisms that determine the transient nature of brusatol׳s inhibitory action toward Nrf2 may provide an opportunity for chemical tuning of the compound, to provide a pharmacological antagonist of Nrf2 with long-lasting effects, so as to minimize the need for repeated dosing in vivo. On the other hand, Zhang and colleagues have reported significant decreases in Nrf2 pathway activity and associated increases in chemotherapeutic efficacy in mice after chronic administration of brusatol [12,14], indicating that the transient nature of brusatol׳s inhibitory effect on Nrf2 signaling can be overcome through repeated dosing in vivo. These findings confirm that brusatol can repress Nrf2 signaling in laboratory animals, whereas we have shown that brusatol can provoke the depletion of Nrf2 in freshly isolated primary human hepatocytes, demonstrating the translational relevance of our detailed investigations in mouse Hepa-1c1c7 hepatoma cells.

In summary, we have shown that brusatol provokes a rapid and transient depletion of Nrf2 protein and consequent downregulation of Nrf2-regulated cell defense processes, thereby sensitizing mammalian cells to chemical stress. Although these findings verify brusatol as a useful experimental tool for the inhibition of Nrf2 signaling, they also highlight the potential for therapeutic inhibition of Nrf2 to alter the risk of adverse events by reducing the capacity of nontarget cells to buffer against chemical and oxidative insults. A current lack of detail makes it difficult to establish the existence of a common mechanism of action for the small number of Nrf2 inhibitors reported to date, including brusatol, retinoic acid receptor α agonists [25], leutolin [26], and trigonelline [27]. The identification of a unifying mechanism would facilitate high-throughput screening of compound libraries, which may reveal novel inhibitors of Nrf2. A better mechanistic understanding would also inform a rational assessment of the risk:benefit ratio of inhibiting Nrf2 in relevant therapeutic contexts, which is essential if compounds such as brusatol are to be developed into efficacious and safe drugs.
