# Supplementary material for: Brusatol provokes a rapid and transient inhibition of Nrf2 signaling and sensitizes mammalian cells to chemical toxicity—implications for therapeutic targeting of Nrf2
Source: Free Radic Biol Med. 2015 Jan;78:202–12. doi: 10.1016/j.freeradbiomed.2014.11.003 (PMC4291150; doi:10.1016/j.freeradbiomed.2014.11.003)
Supplement: Supplementary file 2 — Supplementary Material [file mmc1.docx]

**Brusatol Provokes a Rapid and Transient Inhibition of Nrf2 Signalling and Sensitizes Mammalian Cells to Chemical Toxicity – Implications for Therapeutic Targeting of Nrf2**

Adedamola Olayanju, Ian M. Copple, Holly K. Bryan, George T. Edge, Rowena L. Sison, Min Wei Wong, Zheng-Quan Lai, Zhi-Xiu Lin, Karen Dunn, Christopher M. Sanderson, Ahmad F. Alghanem, Michael J. Cross, Ewa C. Ellis, Magnus Ingelman-Sundberg, Hassan Z. Malik, Neil R. Kitteringham, Christopher E. Goldring, B. Kevin Park.

**Supplementary Material**

**Table S1 – Details of patients donating liver tissue for isolation of primary human hepatocytes.**

| **Donor ID** | **Sex** | **Age** | **Indication** | **Cell Viability (%)** |
| --- | --- | --- | --- | --- |
| PHH1 | Male | 59 | Alcoholic cirrhosis | 81 |
| PHH2 | Female | 74 | Hepatocellular carcinoma | 90 |
| PHH3 | Female | 50 | Colorectal liver metastases | 79 |
| PHH4 | Male | 15 | Crigler-Najjar syndrome type I | 76 |

**Table S2 – Sequences of primers used for RT-qPCR determination of mouse Nrf2, Keap1, Gapdh, Nqo1 and Gclm levels.**

| **Gene** | **Forward Primer (5’ – 3’)** | **Reverse Primer (3’ – 5’)** |
| --- | --- | --- |
| Nrf2 | GACATCCTTTGGAGGCAAGA | AGGCATCTTGTTTGGGAATG |
| Keap1 | CACAGCAGCGTGGAGAGA | CAACATTGGCGCGACTAGA |
| Gapdh | TGTCCGTCGTGGATCTGAC | CCTGCTTCACCACCTTCTTG |
| Nqo1 | AGCGTTCGGTATTACGATCC | AGTACAATCAGGGCTCTTCTCG |
| Gclm | TGACTCACAATGACCCGAAA | GATGCTTTCTTGAAGAGCTTCC |

**Legend for Video 1 – Real-time imaging of Nrf2-Venus in SK-N-AS cells exposed to CDDO-Me and brusatol.** An SK-N-AS cell line, stably expressing Nrf2-Venus under control of the endogenous human Nrf2 promoter, was exposed to 100 nM CDDO-Me at 0 h, followed by 300 nM brusatol at 1 h. Nrf2-Venus expression was recorded at 2 min intervals by confocal microscopy. Video uploaded separately.
